# Factors Associated with First-Time Telehealth Utilization for Marshallese Living in the United States

**DOI:** 10.1089/tmr.2021.0023

**Published:** 2021-10-07

**Authors:** Jennifer A. Andersen, Holly C. Felix, Hari Eswaran, Nalin Payakachat, Don E. Willis, Cari Bogulski, Pearl A. McElfish

**Affiliations:** ^1^ College of Medicine, University of Arkansas for Medical Sciences Northwest, Fayetteville, Arkansas, USA.; ^2^ Fay W. Boozman College of Public Health, University of Arkansas for Medical Sciences, Little Rock, Arkansas, USA.; ^3^ Institute of Digital Health and Innovation, University of Arkansas for Medical Sciences, Little Rock, Arkansas, USA.; ^4^ College of Pharmacy, University of Arkansas for Medical Sciences, Little Rock, Arkansas, USA.; ^5^ Office of Community Health and Research, University of Arkansas for Medical Sciences Northwest, Fayetteville, Arkansas, USA.

**Keywords:** COVID-19, health care disparities, Marshallese, outcome assessment, telemedicine

## Abstract

***Background:*** Mitigation efforts to prevent the spread of COVID-19 included the robust utilization of telehealth. However, racial/ethnic minority populations have demonstrated low telehealth utilization in the past. The aim of this study was to examine the first-time use of telehealth by Marshallese adults during the COVID-19 pandemic, using online survey data collected from 109 Marshallese respondents between July and November of 2020.

***Methods:*** To evaluate the relationships between sociodemographic characteristics, health care access, physical/mental health, and COVID-19-specific measures and the decision to use telehealth, we use bivariate analyses, including *t*-tests and chi-square analysis.

***Results:*** Eighteen respondents (16.5%) indicated they utilized telehealth for the first time during the pandemic. The number of chronic conditions reported was positively associated with the first-time use of telehealth (*p* = 0.013). Although not statistically significant, a higher proportion of Marshallese first-time telehealth users reported limited English proficiency, changes in health status, and changes in health insurance.

***Discussion:*** Although telehealth has been shown to reduce the absolute gaps in health disparities for minority populations, there is limited utilization by Marshallese communities.

***Conclusions:*** Significant research remains on the utilization of telehealth by Marshallese during the COVID-19 pandemic and to increase utilization in the future.

## Introduction

The first cases of COVID-19, the disease caused by a novel coronavirus (SARS-CoV-2), were diagnosed in the United States in early 2020, and the subsequent pandemic disproportionally burdened many racial and ethnic minority groups in the United States.^1–9^ Marshallese, a Pacific Islander subgroup, experienced widespread health and health care disparities before and during the COVID-19 pandemic. Approximately 30,000 Marshallese currently reside in the United States.^10^ In Benton and Washington counties in Arkansas, home to the largest population of Marshallese in the continental United States, Marshallese people represent ∼2.5% of the total population but made up 19% of the COVID-19 cases. The disparities also include risk factors for COVID-19 morbidity and mortality,^11–17^ including a high prevalence of type 2 diabetes (38%) and tuberculosis (19%).^18–23^ The pre-existing health disparities have increased the toll COVID-19 has taken on Marshallese communities in the United States. Between March and June of 2020, 9% of COVID-19 positive Marshallese in these Arkansas counties were hospitalized for COVID-related complications compared with just 1% of cases nationally. Marshallese accounted for 38% of COVID-19 deaths in Benton and Washington counties.^1^

Mitigation efforts to limit the spread of COVID-19 have included finding ways for health care clinics to provide socially distanced care for their patients. One of the outcomes of COVID-19 mitigation efforts was a more robust utilization of telehealth.^24–27^ Prior research has shown telehealth utilization by racial and ethnic minorities is low, and foreign-born, noncitizens, and naturalized citizens in the United States are less likely to use telehealth.^28^ Social and economic barriers to telehealth use for minority groups include a lack of access to broadband internet services and internet capable devices, limited English language proficiency, and the prohibitive cost of telehealth services.^28^ A lack of trust in medical technology, concerns regarding confidentiality and privacy, and the lack of physical presence of a provider in telehealth visits may play a role in the limited utilization of telehealth among many racial and ethnic minority groups.^29–31^

Marshallese individuals are allowed to live and work as “legal non-immigrants” in the United States because of the existing Compact of Free Association (COFA) agreement with the United States; however, many Marshallese residing in Benton and Washington counties have low incomes and lack health insurance.^32,33^ Despite the COFA agreement and a promise by the United States to provide health care to the Marshallese in return for the damages caused by the nuclear bomb testing in the Republic of the Marshall Islands in the 1940s and 1950s, from 1986 until the passage of the Consolidated Appropriations Act in December of 2020, the Marshallese were barred from accessing federal benefits, including Medicaid, thereby increasing the disparities in health care access.^34–36^

Although telehealth services have been more widely available during COVID-19 and have been shown to have potential to reduce health disparities in minority populations, little is known about Marshallese community member use of telehealth. Therefore aim of our study was to examine the first-time use of telehealth by Marshallese adults living in the United States. To do so, we evaluate the relationships between sociodemographic characteristics, health care access and physical/mental health, and COVID-19-specific measures and the use of telehealth. Understanding the social and economic factors, which may act as barriers or facilitators to telehealth utilization in the Marshallese community, is particularly important given the high-risk status of the Marshallese population during the COVID-19 pandemic and the ability of telehealth to reduce disparities in health outcomes for minority populations.

## Methods

The study was approved by the Institutional Review Board at University of Arkansas for Medical Sciences (Protocol No. 261131). Recruitment was conducted in Arkansas by Marshallese community health workers and nationwide through several Facebook pages with a large number of Marshallese followers (e.g., Republic of the Marshall Islands Consulate, Arkansas Coalition of Marshallese, and the Marshallese Educational Initiative). Consent and survey data were documented in Research Electronic Data Capture (REDCap), a web-based software designed for research and data collection and management. The survey utilized a *Completely Automated Public Turing test to tell Computers and Humans Apart* (CAPTCHA) feature to prevent robotic responses. Inclusion criteria specified participants be self-reported Marshallese living in the continental United States and Hawaii and at least 18 years of age. Recruitment took place from July 27, 2020 to November 22, 2020. Participants received a $20 gift card if they completed the survey.

### Measures

Questions from the Behavioral Risk Factor Surveillance System captured demographic information.^37^ Questions from the PhenX toolkit were used to ask other COVID-19 questions.^38^

#### Telehealth utilization

The outcome of interest, telehealth utilizations during the COVID-19 pandemic, was determined by the question “How have you used telehealth?” Telehealth use during the pandemic was defined for respondents in the question prompt as “Telehealth uses video to allow you and your doctor to talk without being in the same room.” Respondents were coded as *nonusers* if they indicated they had not used telehealth during or before the COVID-19 pandemic and *users* if they had indicated using telehealth during the pandemic but not before.

#### Sociodemographic characteristics

Age in years was included as a continuous variable. Birthplace was a categorical variable of being born in the United States, the Marshall Islands, or another location. Many of the sociodemographic questions were dichotomous, including (1) gender of the respondent (male/female), (2) time in the United States (<10 years/≥10 years), (3) marital status (unmarried/un-partnered or married/partnered), (4) education (≤HS/>HS), (5) employment (employed for wages/not employed for wages), and (6) income (≤$30,000/>$30,000).

#### Health care access and physical/mental health

Several variables were dichotomous: (1) current health insurance (yes/no), (2) either a loss or gain of health care coverage during the pandemic, and (3) confidence in medical knowledge (confident/not confident). The number of chronic conditions was a continuous variable with a range of 0–17. Self-rated physical health was a dichotomous variable of excellent/good or fair/poor, and self-rated mental health was a categorical variable of excellent/somewhat good, average, or somewhat poor/poor.^39^

#### COVID-19-specific measures

Variables specific to the COVID-19 pandemic^38^ included (1) a categorical measure of general changes in health during the pandemic (better, worse, or about the same); (2) a count of COVID-19 stressors, including health concerns and access to medical supplies for self or others with a range of 0 to 13; and (3) receipt of a COVID-19 test (yes/no).

### Analysis

Descriptive statistics were calculated to characterize the sample and responses to survey questions, with means and standard deviations for continuous variables and the frequency and percentages for categorical variables. Bivariate statistics (Fisher's exact test or *t*-test) were used to assess the relationship between the decision to utilize telehealth or not and each variable. Analysis was completed using STATA 16,^40^ and *p* ≤ 0.05 was considered statistically significant. In addition, results with *p* ≤ 0.10 were considered approaching significance and are reported due to the small sample size and descriptive nature of the study.

## Results

A total of 120 individuals living in 12 states responded to the survey. States represented include Arizona, Arkansas, California, Hawaii, Michigan, Missouri, Nevada, Oklahoma, Oregon, Tennessee, Texas, and Washington. Of those respondents, 11 (9.2%) indicated they had utilized telehealth before the onset of the COVID-19 pandemic. As our aim was to examine initial use of telehealth during COVID-19, these cases were removed from the sample, leaving a final analytic sample of 109 respondents. Table 1 describes the demographic characteristics of the sample and presents the results of the bivariate tests of association.

**Table 1.** Descriptive Statistics and Bivariate Results for Marshallese Respondents Who Utilized Telehealth During the COVID-19 Pandemic

	Used telehealth during COVID-19		Fisher's exact/t-test *p*
	No (*n* = 91)	Yes (*n* = 18)	
	Mean (SD) or *n* (%)		
Age (*n* = 109)	35.1 (8.5)	36.9 (8.8)	0.401
Gender (*n* = 109)			
Female	58 (63.7)	12 (66.7)	1.00
Male	33 (36.3)	6 (33.3)	
Birthplace (*n* = 109)			
Other	2 (2.2)	0 (0)	1.00
United States	11 (12.1)	2 (11.1)	
Marshall Islands	78 (85.7)	16 (88.9)	
Time in United States (*n* = 107)			
<10 years	31 (34.8)	6 (33.3)	1.00
>10 years	58 (65.2)	12 (66.7)	
English speaking ability (*n* = 109)			
Very well	40 (44.0)	5 (27.8)	*0.075*
Well	43 (47.3)	8 (44.4)	
Not well	8 (8.8)	5 (27.8)	
Marital status (*n* = 108)			
Unmarried/single	20 (22.2)	3 (16.7)	0.758
Married/partnered	70 (77.8)	15 (83.3)	
Education (*n* = 109)			
High school or less	36 (40.4)	7 (38.9)	1.00
Some college or more	53 (59.5)	11 (61.1)	
Employment (*n* = 75)			
Not employed for wages	11 (19.3)	3 (16.7)	1.00
Employed for wages	46 (80.7)	15 (83.3)	
Income (*n* = 99)			
≤$30,000	54 (66.7)	10 (55.6)	0.420
>$30,000	27 (33.3)	8 (44.4)	
Current health insurance (*n* = 101)			
No	34 (40.0)	6 (37.5)	1.00
Yes	51 (60.0)	10 (62.5)	
Change in health insurance during COVID-19 (*n* = 96)			
No change	66 (84.6)	12 (66.7)	*0.104*
Lost health insurance	9 (11.5)	6 (33.3)	
Gained health insurance	3 (3.9)	0 (0)	
Confidence in medical knowledge (*n* = 104)			
Not confident filling out medical forms	26 (30.2)	6 (33.3)	0.785
Confident filling out medical forms	60 (69.8)	12 (66.7)	
No. of chronic conditions (*n* = 83)	0.86 (1.39)	2.5 (4.54)	**0.013**
Self-rated physical health (*n* = 108)			
Excellent/good	72 (80.0)	12 (66.7)	0.225
Fair/poor	18 (20.0)	6 (33.3)	
Self-rated mental health (*n* = 107)			
Excellent/somewhat good	48 (55.3)	8 (47.1)	0.599
Average	33 (36.7)	6 (35.3)	
Somewhat poor/poor	9 (10.0)	3 (17.7)	
General health change during COVID-19 (*n* = 103)			
Better	14 (16.1)	4 (25.0)	*0.092*
Worse	12 (13.8)	5 (31.3)	
About the same	61 (70.1)	7 (43.8)	
COVID-19 Stress Scale (*n* = 109)	6.7 (3.8)	7.0 (3.2)	0.783
Receipt of COVID-19 test (*n* = 101)			
Yes	13 (15.5)	5 (29.4)	0.178
No	71 (84.5)	12 (70.6)	

Significant *p*-values are bolded; trending to significance *p*-values are in italics. Listwise deletion used for missing values.

SD, standard deviation.

### Telehealth utilization

Of the respondents, 91 (83.5%) indicated they did not utilize telehealth, and 18 (16.5%) respondents indicated they utilized telehealth during the COVID-19 pandemic.

### Sociodemographic characteristics

Overall, the results indicate no difference in the utilization of telehealth by any of the sociodemographic characteristics of the respondents.

### Health care access and physical/mental health

The results show the number of chronic conditions (e.g., high blood pressure and type 2 diabetes) reported by the respondent did influence the utilization of telehealth. Respondents who used telehealth reported a mean of 2.5 (±4.54) chronic conditions compared with a mean of 0.86 (±1.39) reported by those who did not use telehealth (*p* = 0.013).

### COVID-19-specific measures

There was not a significant relationship between any of the COVID-19-specific measures and telehealth utilization.

## Discussion

This study explored the relationships between sociodemographic characteristics, health care access, physical/mental health, and COVID-19-specific measures associated with first-time telehealth utilization for Marshallese during the COVID-19 pandemic. Findings show the majority of the Marshallese respondents had not used telehealth before or during the COVID-19 pandemic. This is consistent with previous research that has shown immigrants to the United States, foreign-born noncitizens, and naturalized citizens are less likely to use telehealth; Marshallese are considered “legal non-immigrants” and may face similar social and economic barriers to the use of telehealth.^28,41^ Given the national action plan to increase the use of telehealth in the United States, and in the face of a global pandemic that necessitated the use of telehealth to prevent disease spread, the limited utilization of telehealth is worrisome.^42,43^ The Marshallese face numerous health disparities, and access to regular health care for prevention and control of chronic conditions is imperative in addressing disparate health outcomes in the Marshallese population in the United States.^21,41,44,45^

The only statistically significant relationship to telehealth utilization among the Marshallese respondents was the number of chronic conditions reported. Advancements in telehealth have often focused on the treatment of chronic conditions^46–50^; therefore, this result is not to be unexpected. Throughout the COVID-19 pandemic, many medical specialists have converted to telehealth care to protect their potentially high-risk patients, thereby increasing access to care for those most at risk.^24–26,43^ Given the high rate of diabetes, hypertension, and obesity in the Marshallese population, the risk of adverse outcomes from COVID-19 are high; the move to telehealth among the Marshallese at highest risk is reassuring. In addition, the move to telehealth among the Marshallese with a greater number of diagnosed chronic conditions may help to prevent future non-COVID-related complications. Further study is needed to understand how Marshallese with numerous diagnosed chronic conditions facilitated a successful transition to telehealth, in an effort to improve future engagement of racial and ethnic minorities in these services.

Although not significant, we did see patterns of difference in telehealth utilization by English proficiency during the pandemic. Limited English proficiency (LEP) has been linked to lower engagement with telehealth.^51,52^ Moreover, LEP may be more common among older Marshallese. Given the high prevalence of multigenerational households in the Marshallese community,^53^ there may be younger and/or more English proficient family members who are available to serve as translators during telehealth visits who may not normally be available due to other obligations during regular nonisolating times. Further research will be needed to understand the potential connection between English proficiency and telehealth during the COVID-19 pandemic.

Telehealth utilization also showed potential differences by changes in general health status and changes in health insurance coverage, although they did not reach levels of significance. During the pandemic, screening services for COVID-19 symptoms took place through phone or internet free of charge, which may have encouraged more Marshallese to utilize telehealth services when they had a decline in health status or had no health insurance coverage.

The results should be considered with limitations in mind. The study had a small convenience sample of Marshallese living in the United States, and the education and income level of the sample was higher than other published demographics for Marshallese.^21,54^ Furthermore, the data are cross-sectional, and the utilization of telehealth may change over time as needs and limits on in-person medical visits change. Finally, measures were limited in the questionnaire and did not include measures of internet access, trust in technology, or concerns regarding confidentiality or privacy.

## Conclusions

Despite these limitations, the study makes a significant contribution to the literature. This article is the first to document telehealth utilization among Marshallese living in the United States before or during the COVID-19 pandemic. This is particularly important given the health disparities experienced by the Marshallese. Previous study has indicated that telehealth can narrow the absolute gaps in health outcomes between white and black patients,^55^ yet the results of this study indicate there is a great deal of work to be done in increasing the utilization of telehealth in Marshallese populations.

## Abbreviations Used

CAPTCHA: Completely Automated Public Turing test to tell Computers and Humans Apart
COFA: Compact of Free Association
LEP: limited English proficiency
REDCap: Research Electronic Data Capture
